# A Multi-Server Two-Factor Authentication Scheme with Un-Traceability Using Elliptic Curve Cryptography

**DOI:** 10.3390/s18072394

**Published:** 2018-07-23

**Authors:** Guosheng Xu, Shuming Qiu, Haseeb Ahmad, Guoai Xu, Yanhui Guo, Miao Zhang, Hong Xu

**Affiliations:** 1 School of CyberSpace Security, Beijing University of Posts and Telecommunications, Beijing 100876, China; guoshengxu@bupt.edu.cn (G.X.); xga@bupt.edu.cn (G.X.); yhguo@bupt.edu.cn (Y.G.); zhangmiao@bupt.edu.cn (M.Z.); 2 Elementary Educational College, Jiangxi Normal University, Nanchang 330022, China; 3 Department of Computer Science, National Textile University, Faisalabad 37610, Pakistan; haseeb_ad@hotmail.com; 4 High-Tech Research and Development Center, the Ministry of Science and Technology, Beijing 100044, China; xuhong@htrdc.com

**Keywords:** multi-server, authentication, key agreement, elliptic curve cryptography (ECC), BAN-Logic, wireless sensor networks (WSNs)

## Abstract

To provide secure communication, the authentication-and-key-agreement scheme plays a vital role in multi-server environments, Internet of Things (IoT), wireless sensor networks (WSNs), etc. This scheme enables users and servers to negotiate for a common session initiation key. Our proposal first analyzes Amin et al.’s authentication scheme based on RSA and proves that it cannot provide perfect forward secrecy and user un-traceability, and is susceptible to offline password guessing attack and key-compromise user impersonation attack. Secondly, we provide that Srinivas et al.’s multi-server authentication scheme is not secured against offline password guessing attack and key-compromise user impersonation attack, and is unable to ensure user un-traceability. To remedy such limitations and improve computational efficiency, we present a multi-server two-factor authentication scheme using elliptic curve cryptography (ECC). Subsequently, employing heuristic analysis and Burrows–Abadi–Needham logic (BAN-Logic) proof, it is proven that the presented scheme provides security against all known attacks, and in particular provides user un-traceability and perfect forward security. Finally, appropriate comparisons with prevalent works demonstrate the robustness and feasibility of the presented solution in multi-server environments.

## 1. Introduction

With the recent advancements in Internet and communication technology and the growing demand for sharing multiple data resources, secure and efficient communication between the involved stakeholders has become more essential in areas such as e-commerce, telecare medical information, distributed cloud storage systems, etc. Obviously, privacy protection has emerged as a vital issue for secure and trusted communication. For secure and effective communication over an insecure network, the involved parties are required to negotiate on a common session key beforehand. For such negotiations, authentication-and-key-agreement protocols serve as the only solution. The first password authentication with insecure communication was established by Lamport in 1981 [1]. Later, Frank et al. [2] presented an authentication protocol based on hypertext transport protocol in 1991. However, Yang et al. [3] identified that Frank’s proposal was insecure and provided an improved solution in 2005. In order to present a secure and efficient authentication and key agreement protocol, in the following decade, many single-, two-, and three-factor authentication protocols were constructed while employing RSA, discrete logarithm over general groups, elliptic curve cryptography (ECC), chaotic maps [4,5,6,7,8,9,10,11,12,13,14,15,16,17,18,19,20,21,22], etc. However, some security limitations are prevailing in these protocols. By analyzing a large number of authentication protocols, we found that such shortcomings are resulted due to either improper usage of the cryptographic primitives or design defects of the protocols.

In 2011, Awasthi et al. [23] showed that the protocol of Shen et al. [24] is prone to user impersonation attack. To remedy impersonation attack, Awasthi et al. put forward a refined time stamp-based authentication-and-key-agreement protocol. However, in that protocol, the adversary can easily obtain smart card and identity parameters through an open channel. In 2014, Huang et al. [25] pointed out that the scheme presented by Awasthi et al. is unable to resist against user impersonation attack, and overlooks the password updation stage. Moreover, we remark that Awasthi et al.’s scheme also fails to ensure user anonymity. Huang et al. proposed an enhanced time stamp-based two-factor remote user authentication protocol while incorporating RSA, and claimed that the scheme can resist various attacks. However, Amin et al. [26] proved that the proposal of Huang et al., is susceptible to impersonation, offline password guessing, and insider attacks, while also having an inefficient password updation stage. Keeping in view the limitations of Huang et al.’s proposal, Amin et al. presented an authentication-and-key-agreement mechanism based on RSA.

**Figure 1:**
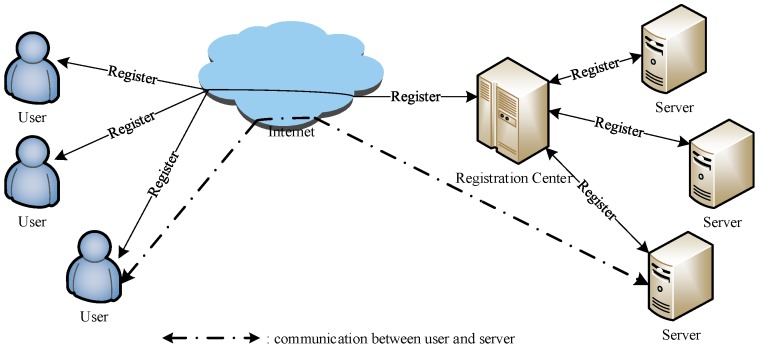
The architecture of the multi-server authentication system.

In a multi-server environment, users interact with multiple servers. To login with different identities and passwords in such an environment is troublesome for the users. To eliminate this problem, first, users and multiple servers are registered at the registration center (RC). Subsequently, users can make an authentication-and-key-agreement with multiple servers by utilizing the unique identity and password pair. A proposed architecture of the multi-server authentication system is depicted in Figure 1. In 2013, Pippel et al. [27] employed smart cards to present a robust multi-server authentication protocol and proved it to be resistant against various known attacks. In a subsequent work, Li et al. [28] identified that the protocol presented by Pippel et al. is unable to provide correct authentication. Moreover, it cannot withstand impersonation attack and insider attack. Afterwards, Li et al. designed an improved smart card authentication protocol and proved that it can withstand perfect forward secrecy, stolen smart-card attack, offline password guessing attack, and so on. Even so, Srinivas et al. [29] provided that Li et al.’s scheme is unable to resist insider attack, denial-of-service attack, and stolen smart-card attack, and cannot provide perfect forward secrecy. However, we remark that Li et al.’s scheme addresses perfect forward secrecy. As a solution, Srinivas et al. presented an improved two-factor authentication scheme for the same multi-server architecture with reduced computation and communication cost while claiming that their protocol is susceptible to various known attacks. To the best of our knowledge, most of the schemes cannot provide perfect forward secrecy and user un-traceability, and are susceptible to key-compromise user impersonation attack and offline password guessing attack. More precisely, once an authentication-and-key-agreement mechanism fails to ensure user un-traceability, the user’s entire whereabouts are exposed to the attacker. This provides a great deal of convenience for attackers to carry out more attacks. This proposal takes the schemes of Amin et al. and Srinivas et al. as examples to depict how an adversary traces the legal user, effectively guesses the correct password, or succeeds in obtaining the session key. These security flaws usually exist in wireless sensor networks (WSNs) as well [30,31,32,33,34,35,36,37,38,39,40]. Moreover, The methods of attacking and designing we use are very useful and effective in analyzing similar vulnerabilities and designing new protocols in WSNs, respectively.

### 1.1. Contributions

The key contributions of our proposal are listed as follows: (1) We prove that Amin et al.’s protocol fails to ensure perfect forward secrecy and user un-traceability, and is susceptible to key-compromise user impersonation attack and offline password guessing attack. (2) It is proven that Srinivas et al.’s scheme fails to ensure user un-traceability, and is prone to key-compromise user impersonation attack and offline password guessing attack. (3) To overcome these limitations, we design a two-factor authentication-and-key-agreement scheme for multi-server architecture while incorporating ECC. (4) The presented scheme ensures perfect forward secrecy, user anonymity, and un-traceability. Moreover, it provides security against major attacks, including impersonation attack, offline password guessing attack, key-compromise user impersonation attack, etc. (5) The security analysis using Burrows–Abadi–Needham logic (BAN-Logic) provides that the proposed protocol ensures secured mutual authentication between a remote user and server.

### 1.2. Outline of This Paper

The remaining contents of the proposal are organized as follows: cryptographic primitive and attacker model are detailed in Section 2. The scheme of Amin et al., and its cryptanalysis are presented in Section 3 and Section 4, respectively. Section 5 and Section 6 provide the scheme of Srinivas et al., and its cryptanalysis, respectively. The improved version of the proposed scheme is provided in Section 7. The heuristic security analysis and BAN-Logic are presented in Section 8 and Section 9, respectively. Section 10 details the security and performance comparisons. Finally, Section 11 contains the concluding remarks.

## 2. Preliminary

We take advantage of ECC to present a two-factor authentication scheme. The following section briefly introduces the collision-resistant cryptographic one-way hash function as well as some computationally infeasible problems, including the elliptic curve computational Diffie–Hellman Problem (ECCDHP) and the elliptic curve discrete-logarithm problem (ECDLP). Table 1 depicts some notations and descriptions that are used in the proposed scheme.

### 2.1. Collision-Resistant One-Way Hash Function

Basically, the one-way hash function $H\left(\cdot \right):{\left\{0,1\right\}}^{*}\to {\left\{0,1\right\}}^{n}$ requires an input in the form of an arbitrary length binary string $x\in {\left\{0,1\right\}}^{*}$, and yields a string in binary form $y=H\left(x\right)\in {\left\{0,1\right\}}^{n}$. In brief terms, a cryptographic collision-resistant one-way hash function H(·) ensures the following:

1. Given $y\in {\left\{0,1\right\}}^{n}$, it is difficult to determine the input $x\in {\left\{0,1\right\}}^{*}$ within polynomial time.
2. It is difficult to determine $x^{\prime }\in {\left\{0,1\right\}}^{*}$ such that $H\left(x\right)=H\left(x^{\prime }\right)$, where $x^{\prime }\neq x$.
3. It is difficult to uncover a pair $\left(x,x^{\prime }\right)\in {\left\{0,1\right\}}^{*}$, such that $x^{\prime }\neq x$ and $H\left(x\right)=H\left(x^{\prime }\right)$ could hold.

### 2.2. Intractable Problems in ECC

The elliptic curve equation over a finite field $F_{p}$ in ECC takes the form $E_{p}\left(a,b\right):y^{2}=x^{3}+ax+b$(modp), where $4a^{3}+27b\neq 0$(modp) and $a,b\in F_{p}$ [41].

1. *ECDLP*: The elliptic curve discrete-logarithm problem over elliptic curve $E_{p}\left(a,b\right)$ refers to computing $m\in F_{p}^{*}$ from Q=mP for given $P,Q\in E_{p}\left(a,b\right)$.
2. *ECCDHP*: The elliptic curve computational Diffie–Hellman problem over elliptic curve $E_{p}\left(a,b\right)$ refers to computing mnP, given points $mP,nP\in E_{p}\left(a,b\right)$.

### 2.3. Adversary Model

According to [18,42,43,44,45,46,47], the capacities of A in authentication and key agreement schemes, which are used in cryptanalysis of Amin et al.’s scheme, Srinivas et al.’s scheme, and our proposed scheme, are listed as follows:

1. A is able to intercept, block, delete, modify, and resend the message contents through an open channel.
2. Because identity and password have low entropy, A can enlist all pairs of $\left(Pw_{i},Id_{i}\right)$ simultaneously from $\left(D_{Pw},D_{Id}\right)$ within polynomial time, where $D_{Pw}$ and $D_{Id}$ refer to the space of passwords and identities in $D_{Pw}$ and $D_{Id}$, respectively.
3. A can either acquire $Pw_{i}$ of the $U_{i}$ via malicious device or reveal the information from SC, but is not permitted to use both methods together.
4. A can acquire a server’s private key while evaluating forward secrecy or key-compromise user impersonation attack.
5. A has the ability to reveal all parameters of the smart card when assessing stolen smart-card attack, offline password guessing attack, impersonation attack, forward secrecy, etc.

## 3. Brief Review of Amin et al.’s Proposal

This section provides a brief review of Amin et al.’s [26] authentication scheme for Session Initiation Protocol (SIP). The scheme presented by the authors comprises four stages: initialization, registration, login and authentication, and password updation. We omit the description of the password updation stage.

### 3.1. Initialization

*S* takes two large primes *p* and *q* as secret parameters to calculate n=p×q as a public parameter. Afterwards, *S* chooses a prime *e* to obtain *d* by computing e×d≡1mod(p−1)(q−1), such that 1<e<(p−1)(q−1).

### 3.2. Registration

1. $U_{i}$ enters an identity $Id_{i}$ and password $Pw_{i}$. Subsequently, $U_{i}$ randomly picks up a number *r* and calculates $PWr_{i}=H(Pw_{i}\left||u\right)$. Afterwards, $U_{i}$ transmits the registration request message $\left\{Id_{i},PWr_{i}\right\}$ to *S* via secure medium.
2. Upon receiving the request message $\left\{Id_{i},PWr_{i}\right\}$ from the new user $U_{i}$, *S* calculates $CId_{i}=H(Id_{i}\left||d\right)$, $Reg_{i}=H(CId_{i}||PWr_{i}||Id_{i})$, and $Y_{i}=CId_{i}⊕H(PWr_{i}||Id_{i})$. Afterwards, *S* stores the contents $\left\{Reg_{i},Y_{i},n,e,H\left(\cdot \right)\right\}$ in a new card SC and sends SC to $U_{i}$.
3. Once obtaining SC, $U_{i}$ stores *u* into SC.

### 3.3. Login and Authentication

1. To start the session with the *S*, $U_{i}$ inserts SC into a card reader and inputs their login details, including $Id_{i}$ and $Pw_{i}$. Subsequently, SC calculates $PWr_{i}=H(Pw_{i}||r),CId_{i}=Y_{i}⊕H(PWr_{i}||Id_{i})$, and $Reg_{i}=H(DId_{i}||PWr_{i}||Id_{i})$. Afterwards, it verifies the value of $Reg_{i}$. In case of invalid values, the session is ended. Otherwise, SC randomly chooses a number $N_{1}$, the current time stamp $T_{u}$, and calculates $D_{i}=H(CId_{i}||H(PWr_{i}||Id_{i})||T_{u}\left|\right|N_{1})$ and $L_{i}=(Id_{i}\left|\right|D_{i}\left|\right|N_{1}{)}^{e}\;\mathrm{mod}\;n$. Next, SC transmits the login request message $\left\{L_{i},Y_{i},T_{u}\right\}$ to *S*.
2. Upon receiving the login request from $U_{i}$, *S* verifies the time stamp $T_{u}$ corresponding to the current time stamp $T_{s}$. In the case of valid time stamp $T_{u}$, it continues to execute the following steps. Otherwise, it aborts the session. Afterwards, *S* decrypts $L_{i}$ to obtain $\left(Id_{i}^{*}||D_{i}^{*}||N_{i}^{*}\right)$ and then checks whether $CId_{i}^{*}=H(Id_{i}^{*}||d),H(PWr_{i}||Id_{i}{)}^{*}=Y_{i}⊕CId_{i}^{*}$ and $D_{i}^{**}=H(CId_{i}^{*}||H(PWr_{i}||Id_{i}{)}^{*}\left|\right|T_{u}\left|\right|N_{1}^{*})$. Afterwards, *S* checks $D_{i}^{**}=?D_{i}^{*}$. After finishing this verification, *S* randomly selects a number and computes $X_{i}=H(N_{2}||CId_{i}),Z_{i}=N_{i}⊕N_{2}$. Finally, *S* transmits the respond message $\left\{X_{i},Z_{i},T_{s}\right\}$ to SC via public channel.
3. Once receiving the response message from *S*, SC checks the validity of $T_{s}$. After finishing the verification, SC checks whether $N_{2}^{*}=N_{1}⊕Z_{i},X_{i}^{*}=H(N_{2}^{*}||CId_{i})$ and verifies $X_{i}^{*}=?X_{i}$. If it holds, $U_{i}$ accepts the response message. Finally, *S* and $U_{i}$ calculate the session key: $SK=H(N_{1}||CId_{i}\left|\right|N_{2}^{*})=H(N_{i}^{*}||CId_{i}^{*}\left|\right|N_{2}).$

## 4. Limitations of Amin et al.’s Scheme

According to the adversary model presented in Section 2.3, in the following, we prove that Amin et al.’s scheme is unable to provide user un-traceability and perfect forward secrecy, and is prone to key-compromise user impersonation attack and offline password guessing attack.

### 4.1. User Un-Traceability

Observing the protocol of Amin et al., it can be found that $Y_{i}$ is transmitted during the login request message stage. However, $Y_{i}=CID_{i}⊕h(PWr_{i}||ID_{i})$ is a fixed value in SC, unless $U_{i}$ changes their password during the password updation stage. Usually, the user does not change their password after every session. Therefore, $U_{i}$ can be traced by the adversary using $Y_{i}$. Hence, Amin et al.’s protocol does not ensure user un-traceability.

### 4.2. Offline Password Guessing Attack

Offline password guessing attack is the main limitation for most of the presented proposals addressing authentication. If A somehow steals the SC of $U_{i}$ and embeds the data $\left\{Reg_{i},Y_{i},r\right\}$ in it, then the adversary A can perform the following steps to obtain $Id_{i}$ and $Pw_{i}$ of $U_{i}$.

1. From the password dictionary space $D_{PW}$, the adversary A randomly chooses the password $PW^{*}$, and picks up the identity $ID^{*}$ from the identity dictionary space $D_{ID}$.
2. A calculates $PWr_{i}^{*}=h(Pw^{*}\left||r\right)$.
3. A calculates $CID_{i}^{*}=Y_{i}⊕h(PWr_{i}^{*}||ID_{i}^{*})$.
4. A calculates $Reg_{i}^{*}=h(CID_{i}^{*}||PWr_{i}^{*}||ID_{i}^{*})$.
5. To check the correctness of $Pw^{*}$ and $Id^{*}$, A examines whether $Reg_{i}^{*}=Reg_{i}$, where $Reg_{i}$ belongs to SC of $U_{i}$.
6. If the aforementioned equality holds, A’s guess results as successful. Otherwise, A repeats Steps 1–5 until it obtains the correct password and identity of $U_{i}$.

From the aforementioned procedure, we find that the computational time complexity of offline password guessing attack is $O(|D_{PW}|*|D_{ID}|*3T_{h}),$ where $|D_{Pw}\left|,\right|D_{Id}|$, and $T_{h}$ refer to the number of $D_{Pw}$, the number of $D_{Id}$, and the performing time of hash function h(·), respectively. According to [48,49,50], usually, $|D_{Id}\left|<\right|D_{Pw}|<10^{6}$. Therefore, the aforementioned attack is very efficient. Hence, Amin et al.’s protocol is unable to resist offline password guessing attack. Actually, the verified data $Reg_{i}$ are stored in $U_{i}$’s smart card, which is the main reason for the success of the above attack. By computing $Reg_{i}$, the smart card is able to check the correct login of the legal user. Moreover, it also gives A the chance to guess password and identity. Since the identity and password have low entropy in such scenarios, A can guess them successfully within polynomial time.

### 4.3. Lacks of Perfect Forward Secrecy

Assume that the A obtains the long term private key *d* of *S* and eavesdrops the transmitted message $\left\{L_{i},Y_{i},T_{u}\right\},\left\{X_{i},Z_{i},T_{s}\right\}$. Having that information, A can easily calculate two key random numbers $\left\{N_{1},N_{2}\right\}$. A undergoes the following procedure to compute SK between $U_{i}$ and *S*.

1. The adversary A computes ${\left(L_{i}\right)}^{d}\;\mathrm{mod}\;n=(ID_{i}\left|\right|D_{i}\left|\right|N_{1})$ to obtain $\left\{ID_{i},N_{1}\right\}$.
2. A computes $CID_{i}=h(ID_{i}\left||d\right)$.
3. A computes $N_{2}=Z_{i}⊕N_{1}$.
4. A computes $SK=h(N_{1}||CID_{i}||N_{2})$.

The computational time overhead of the aforementioned attack is $O(2T_{h}+T_{e}+T_{eor})$, where $T_{e}$ and $T_{eor}$ are the running time of modular exponentiation and exclusive-or operation, respectively. Therefore, the protocol of Amin et al. does not ensure perfect forward secrecy. This problem can be solved by adding an operation of public key cryptography, which slightly increases the computation load. However, it is a feasible approach in terms of the trade-off between security and practicality.

### 4.4. Key-Compromise User Impersonation Attack

If the long-term private key *d* of *S* is revealed to the adversary A in Amin et al.’s protocol, A can impersonate the legitimate user $U_{i}$ to *S* as follows:

1. A computes ${\left(L_{i}\right)}^{d}\;\mathrm{mod}\;n=(Id_{i}\left|\right|D_{i}\left|\right|N_{1})$, and subsequently calculates $CId_{i}=H(Id_{i}\left||d\right)$ and $A=H(PWr_{i}||Id_{i})=Y_{i}⊕CId_{i}$.
2. A obtains the login request message $\left\{L_{i},Y_{i},T_{u}\right\}$ of $U_{i}$, randomly selects a number $N_{a}$, and computes $D_{i}^{\prime }=H(CId_{i}\left||A|\right|T_{u}^{\prime }\left|\right|N_{a}),L_{i}^{\prime }=(Id_{i}\left|\right|D_{i}^{\prime }\left|\right|N_{a}{)}^{e}\;\mathrm{mod}\;n.$ Afterwards, A transmits the forged request message $\left\{L_{i}^{\prime },Y_{i},T_{u}^{\prime }\right\}$ to *S*.
3. Upon receiving the forged message, obviously *S* can verify it successfully. Thus, *S* randomly provokes a number $N_{2}^{\prime }$, and computes $X_{i}^{\prime }=H(N_{2}^{\prime }||CId_{i})$ and $Z_{i}^{\prime }=N_{a}⊕N_{2}^{\prime }$. Finally, *S* sends $\left\{X_{i}^{\prime },Z_{i}^{\prime },T_{s}\right\}$ to A.
4. Upon receiving the response from *S*, A calculates $N_{2}^{\prime }=N_{a}⊕Z_{i}^{\prime }$. Finally, the server *S* believes that $SK=H(N_{a}||CId_{i}||N_{2})$ is the common session key between a legitimate user and itself. However, in actual terms, A acts as $U_{i}$.

Therefore, Amin et al.’s protocol is unable to resist key-compromise user impersonation attack.

## 5. Review of Srinivas et al.’s Scheme

The following section reviews Srinivas et al.’s protocol [29] comprising four steps: initialization, registration, login and authentication, and password updation stage.

### 5.1. Initialization

The trusted registration center RC during this stage selects a 1024-bit large prime *p*, generates $g\in Z_{p}^{*}$, chooses a one-way hash function $H\left(\cdot \right):{\left\{0,1\right\}}^{*}\to Z_{p}^{*}$, and randomly picks a number mk as the master secret key.

### 5.2. Registration Process

#### 5.2.1. Server Registration

$S_{j}\left(1\leq j\leq k\right)$ chooses a unique identity $SId_{j}$ and sends $SId_{j}$ to RC through a secure-medium. Upon receiving $SId_{j}$, RC calculates $r_{j}=H(SId_{j}\left||mk\right)$, and sends $\left\{r_{j},p,g,H\left(\cdot \right)\right\}$ to $S_{j}$ through a secure medium.

#### 5.2.2. User Registration

First, a new user $U_{i}$ selects $Id_{i}$, $Pw_{i}$, and randomly chooses a number $r_{i}$. Subsequently, the user calculates $UId_{i}=H(Id_{i}\left|\right|r_{i}),RPw_{i}=H(Pw_{i}\left|\right|r_{i})$ and sends $\left\{UId_{i},RPw_{i}\right\}$ to RC. Upon receiving the registration request, RC calculates $v_{ij}=H(r_{j}||UId_{i}),s_{ij}=v_{ij}⊕RPw_{i}.$ Afterwards, RC sends $U_{i}$ a new smart card $SC_{i}$ containing $\left\{s_{i1},s_{i2},\cdots ,s_{ik},p,g,H\left(\right)\right\}$ through a secure medium. Finally, upon receiving $SC_{i}$ from RC, $U_{i}$ inputs $B_{i}=r_{i}⊕H(Id_{i}||Pw_{i})$ to $SC_{i}$.

### 5.3. Login and Authentication

1. $U_{i}$ inserts $SC_{i}$ into a card reader and inputs $Id_{i}$ and $Pw_{i}$. $SC_{i}$ checks $r_{i}=B_{i}⊕H(Id_{i}||Pw_{i})$, $UId_{i}=H(Id_{i}\left||n\right)$ and $RPw_{i}=H(Pw_{i}\left|\right|r_{i})$. Afterwards, $SC_{i}$ randomly generates a number *a*, chooses the current time stamp $T_{i}$, and calculates $X_{i}=g^{a}\;\mathrm{mod}\;p,v_{ij}=s_{ij}⊕H(UId_{i}||RPw_{i})$ and $h_{ij}=H(v_{ij}||UId_{i}||SId_{j}\left|\right|T_{i}\left|\right|X_{i})$. Subsequently, $SC_{i}$ transmits the login request message $\left\{UId_{i},X_{i},h_{ij},T_{i}\right\}$ to $S_{j}$.
2. $S_{j}$ receives the request message from $U_{i}$, figures out $h_{ij}^{*}=H(H(r_{j}||UId_{i})||UId_{i}||SId_{j}\left|\right|X_{i}\left|\right|T_{i})$, and checks $h_{ij}^{*}=?h_{ij}$. $S_{j}$ terminates the login request if the expression does not hold. Apart from that, $S_{j}$ a random number *b* and calculates $Y_{j}=g^{b}\;\mathrm{mod}\;p,z_{ji}={\left(X_{i}\right)}^{b}\;\mathrm{mod}\;p$. Afterwards, $S_{j}$ picks the current time stamp $T_{j}$ and computes $SK_{ji}=H(UId_{i}||SId_{j}\left|\right|T_{i}\left|\right|h_{ij}^{*}\left|\right|T_{j}\left|\right|z_{ji})$ and $R_{j}=H(UId_{i}\left|\right|T_{i}||H(r_{j}||UId_{i})||T_{j}||SK_{ji}\left|\right|Y_{j})$. Finally, $S_{j}$ sends the response message $\left\{Y_{j},R_{j},T_{j}\right\}$ to $SC_{i}$.
3. On receiving the response message, $SC_{i}$ figures out $z_{ij}={\left(Y_{j}\right)}^{a}\;\mathrm{mod}\;p,$$SK_{ij}=H(UId_{i}||SId_{j}\left|\right|T_{i}\left|\right|h_{ij}\left|\right|T_{j}\left|\right|z_{ji})$, and $R_{j}^{*}=H(UId_{i}\left|\right|T_{i}\left|\right|v_{ij}\left|\right|T_{j}||SK_{ij}\left|\right|Y_{j}).$ Subsequently, $SC_{i}$ checks $R_{j}^{*}=?R_{j}$ and terminates this login request if the expression does not hold. Otherwise, $SC_{i}$ calculates $R_{i}=H(UId_{i}\left|\right|X_{i}\left|\right|Y_{j}||SK_{ij}\left|\right|v_{ij})$ and transmits it to $S_{j}$ through a public channel.
4. Upon acquiring $R_{i}$, $S_{j}$ computes $R_{i}^{*}=H(UId_{i}\left|\right|X_{i}\left|\right|Y_{j}||SK_{ji}||H(r_{j}||UId_{i}))$ and checks $R_{i}^{*}=?R_{i}$. After successful accomplishment of all steps, $S_{j}$ and $U_{i}$ believe that they have the common session key $SK_{ij}=SK_{ji}$.

### 5.4. Password Updation Stage

After the authentication session between $SC_{i}$ and targeted server $S_{j}$, $U_{i}$ inputs $Id_{i},Pw_{i}$, and a new password $Pw^{new}$. Subsequently, $SC_{i}$ calculates $B_{i}^{new}=r_{i}⊕H(Id_{i}||Pw_{i}^{new})$ and $s_{ij}^{new}=s_{ij}⊕H(UId_{i}||H(Pw_{i}\left|\right|r_{i}))⊕H(UId_{i}||H(Pw_{i}^{new}\left|\right|r_{i}))$, where 1≤j≤k. Afterwards, $SC_{i}$ replaces $\left\{s_{i1},s_{i2},\cdots ,s_{ik},B_{i}\right\}$ with $\{s_{i1}^{new},s_{i2}^{new},\cdots ,s_{ik}^{new},B_{i}^{new}\}.$

## 6. Limitations of Srinivas et al.’s Protocol

According to the adversary model presented in Section 2.3, we present some possible attacks for Srinivas et al.’s protocol, including key-compromise user impersonation attack, offline password guessing attack, and lack of user un-traceability. The details are described in the following sections.

### 6.1. Offline Password Guessing Attack

Assume that A extracts the information $\left\{s_{i1},s_{i2},\cdots ,s_{ik},B_{i},p,g,H\left(\cdot \right)\right\}$ of $SC_{i}$ by side-channel attack. Now, A can execute the following steps to get the correct identity $ID_{i}$ and password $PW_{i}$ of user $U_{i}$ in polynomial time.

1. From the password dictionary space $D_{PW}$, the adversary A chooses the password $PW^{*}$, and picks up the identity $Id^{*}$ from the identity dictionary space $D_{Id}$.
2. A computes $n^{*}=B_{i}⊕H(Id_{i}||Pw_{i})$.
3. A computes $RPw_{i}=H(Pw_{i}\left|\right|n^{*})$.
4. A computes $v_{ij}^{*}=s_{ij}⊕H(UId_{i}||RPw_{i})$.
5. A computes $h_{ij}^{*}=H(v_{ij}^{*}||UId_{i}||SId_{j}\left|\right|X_{i}\left|\right|T_{i})$.
6. A verifies whether $h_{ij}^{*}=h_{ij}$, where $h_{ij}$ is acquired from smart card of $U_{i}$.
7. If it holds, then $Pw^{*}$ and $Id^{*}$ is the correct identity and password pair. Otherwise, A repeats Steps 1–6 until it obtains the correct identity and password of $U_{i}$.

We determine the computational time complexity of the aforementioned attack algorithm. That is,

$$O(|D_{Pw}|*|D_{Id}|*4T_{h}),$$

where $|D_{Pw}\left|,\right|D_{Id}|$, and $T_{h}$ are the number of $D_{Pw}$, the number of $D_{Id}$, and the time to compute hash function h(·), respectively. According to [48,49,50], usually, $|D_{Id}\left|<\right|D_{Pw}|<10^{6}$. Therefore, the offline password guessing attack is very efficient. Thus, Srinivas et al.’s protocol is not resistant against offline password guessing attack.

### 6.2. Lack of User Un-Traceability

It can be observed from Srinivas et al.’s protocol that the attacker can get $UId_{i}$ transmitted within the login request message. Since $UId_{i}=H(Id_{i}\left|\right|r_{i})$ is a fixed value, where $Id_{i}$ and $r_{i}$ are invariable, unless the user $U_{i}$ changes their password during the password updation stage, any adversary can trace the user $U_{i}$ by using $UId_{i}$. Therefore, Srinivas et al.’s protocol cannot provide user un-traceability.

### 6.3. Key-Compromise User Impersonation Attack

If the long-term private key $r_{j}$ of $S_{j}$ is revealed to A in Srinivas et al.’s protocol, then A can adopt the following actions to impersonate the legitimate $U_{i}$ to $S_{j}$.

1. A intercepts the login request message $\left\{UId_{i},X_{i},h_{ij},T_{i}\right\}$ of $U_{i}$, and calculates $v_{ij}=H(r_{j}||UId_{i})$.
2. A randomly selects a number $a^{\prime }$ to compute $X_{i}^{\prime }=g^{a^{\prime }}\;\mathrm{mod}\;p,h_{ij}^{\prime }=H(v_{ij}||UId_{i}||SId_{j}\left|\right|X_{i}^{\prime }\left|\right|T_{i}^{\prime })$. Afterwards, A sends the forged login request message $\left\{UId_{i},X_{i}^{\prime },h_{ij},T_{i}^{\prime }\right\}$ to $S_{j}$.
3. Obviously, the forged message can pass the verification of $S_{j}$. Thus, $S_{j}$ randomly chooses a number $b^{\prime }$ to compute $Y_{j}^{\prime }=g^{b^{\prime }}\;\mathrm{mod}\;p,$$z_{ji}^{\prime }={\left(X_{i}^{\prime }\right)}^{b^{\prime }}\;\mathrm{mod}\;p$. Subsequently, $S_{j}$ chooses the current time stamp $T_{j}^{\prime }$ to compute $SK_{ji}^{\prime }=H(UId_{i}||SId_{j}\left|\right|T_{i}^{\prime }\left|\right|h_{ij}^{\prime }\left|\right|T_{j}^{\prime }\left|\right|z_{ji}^{\prime })$ and $R_{j}^{\prime }=H(UId_{i}\left|\right|T_{i}^{\prime }||H(r_{j}||UId_{i})||T_{j}^{\prime }||SK_{ji}^{\prime }\left|\right|Y_{j}^{\prime })$. Finally, $S_{j}$ sends the response message $\left\{Y_{j}^{\prime },R_{j}^{\prime },T_{j}^{\prime }\right\}$ to A.
4. On receiving the response message, A figures out $z_{ij}^{\prime }={\left(Y_{j}^{\prime }\right)}^{a^{\prime }}\;\mathrm{mod}\;p,SK_{ij}^{\prime }=H(UId_{i}||SId_{j}\left|\right|T_{i}^{\prime }\left|\right|h_{ij}^{\prime }\left|\right|T_{j}^{\prime }\left|\right|z_{ij}^{\prime }).$ Subsequently, A calculates $R_{i}^{\prime }=H(UId_{i}\left|\right|X_{i}^{\prime }\left|\right|Y_{j}^{\prime }||SK_{ij}^{\prime }\left|\right|v_{ij}^{\prime })$ and transmits it to $S_{j}$ through a public channel.
5. $S_{j}$ receives $R_{i}^{\prime }$, computes $R_{i}^{\prime \prime }=H(UId_{i}\left|\right|X_{i}^{\prime }\left|\right|Y_{j}^{\prime }||SK_{ji}^{\prime }||H(r_{j}||UId_{i}))$, and checks whether $R_{i}^{\prime \prime }=?R_{i}^{\prime }$. After finishing all steps successfully, $S_{j}$ believes that it holds the common session key $SK_{ij}^{\prime }=SK_{ji}^{\prime }$ with $U_{i}$. Actually, however, A plays as $U_{i}$. Thus, A successfully impersonated $U_{i}$ to $S_{j}$ under the condition that the long-term private key of the server was leaked.

Therefore, Srinivas et al.’s protocol is prone to key-compromise user impersonation attack.

## 7. The Improved Scheme

**Figure 2:**
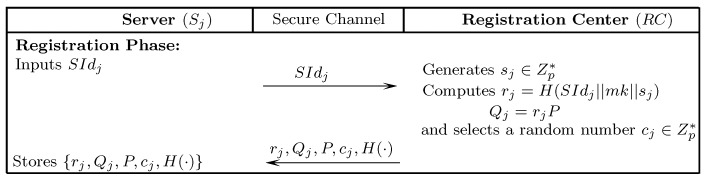
Server registration.

**Figure 3:**
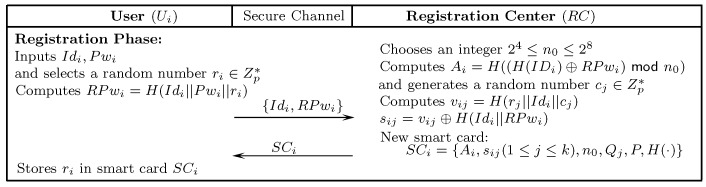
User registration.

**Figure 4:**
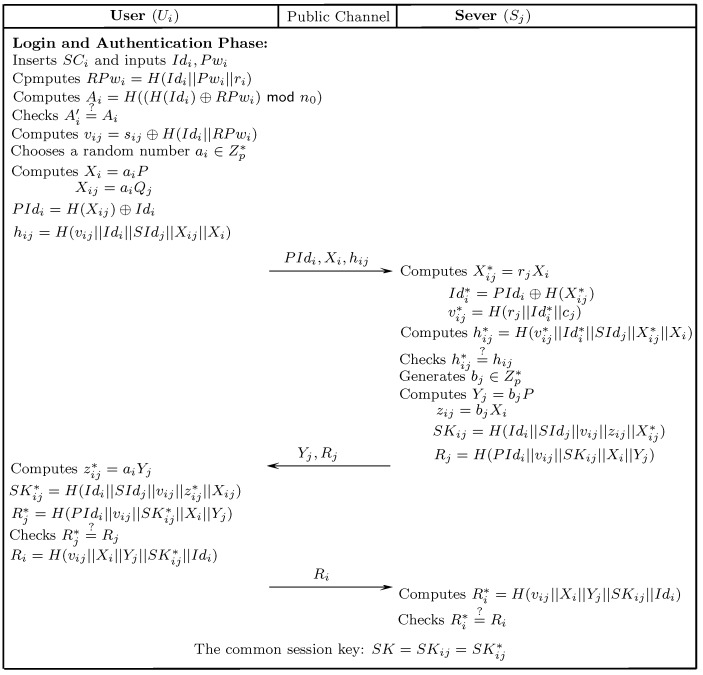
Login and authentication.

The following section presents an improved mutual authentication protocol that gets motivation from Srinivas et al.’s [29] scheme to incorporate ECC. The presented solution not only remedies the limitations of Amin et al.’s [26] and Srinivas et al.’s [29] schemes, but also ensures mutual authentication and is resistant to many known attacks. The presented scheme comprises five stages: initialization, server registration, user registration, authentication-and-key-agreement, and password updating. The notations of the presented scheme are listed in Table 1. Figure 2, Figure 3 and Figure 4 depict the registration and authentication process of the proposed protocol.

### 7.1. Initialization

RC chooses an elliptic curve $E_{p}\left(a,b\right)$ from $F_{p}$, where *p* is a 160-bit-long prime number. Afterwards, RC selects a fixed point $P\neq \infty \in E_{p}\left(a,b\right)$, and one-way hash function $H\left(\right):{\left\{0,1\right\}}^{*}\to Z_{p}^{*}$, and randomly picks a number as mk.

### 7.2. Server Registration

1. $S_{j}$ chooses an identity $SId_{j}$ and transmits it to RC via a secure-medium.
2. RC receives the registration message, randomly generates a number $s_{j}\in Z_{p}^{*}$, and computes $r_{j}=H(SId_{j}\left||mk|\right|s_{j}),Q_{j}=r_{j}P.$ Subsequently, RC randomly generates a number $c_{j}$ for $S_{j}$. Finally, RC sends $\left\{r_{j},Q_{j},c_{j},P,H\left(\cdot \right)\right\}$ to $S_{j}$ through secure-medium.
3. $S_{j}$ stores $\left\{r_{j},Q_{j},c_{j},P,H\left(\cdot \right)\right\}$ in its database.

### 7.3. User Registration

After the successful registration of $U_{i}$ with RC, $U_{i}$ can communicate with any server $S_{j}\left(1\leq j\leq k\right)$.

1. $U_{i}$ selects $Id_{i}$, $Pw_{i}$, and randomly generates a number $r_{i}\in Z_{p}^{*}$ to compute $RPw_{i}=H(Id_{i}||Pw_{i}\left|\right|r_{i})$. Afterwards, $U_{i}$ transmits the registration request message $\left\{Id_{i},RPw_{i}\right\}$ to RC through a secure medium.
2. Upon receiving the registration message, RC randomly generates numbers $r_{s}\in Z_{p}^{*},2^{4}\leq n_{0}\leq 2^{8}$, and computes the following: $A_{i}=H\left(\left(H\left(Id_{i}\right)⊕RPw_{i}\right)\;\mathrm{mod}\;n_{0}\right),$$v_{ij}=H(r_{j}||Id_{i}\left|\right|c_{j}),$$s_{ij}=v_{ij}⊕H(Id_{i}||RPw_{i})$, where (1≤j≤k). Afterwards, RC inserts $\{A_{i},s_{ij}\left(1\leq j\leq k\right)$, $n_{0},Q_{j},P,H\left(\cdot \right)\}$ into a new $SC_{i}$. and sends it to $U_{i}$ through secure-medium.
3. $U_{i}$ stores $r_{i}$ in $SC_{i}$.

### 7.4. Login and Mutual Authentication

$U_{i}$ initiates the login and authentication request for sending to $S_{j}$ by performing the following steps.

1. $U_{i}$ inserts $SC_{i}$ into a card reader and inputs $Id_{i}$, $Pw_{i}$. $SC_{i}$ computes $RPw_{i}=H(Id_{i}||Pw_{i}\left|\right|r_{i})$, and subsequently calculates $A_{i}^{*}=H\left(\left(H\left(Id_{i}\right)⊕RPw_{i}\right)\;\mathrm{mod}\;n_{0}\right)$. Afterwards, $SC_{i}$ inspects the correctness of $A_{i}^{*}$ while comparing it with the value of $A_{i}$ sorted in $SC_{i}$. If $A_{i}^{*}=A_{i}$, $Id_{i}$ and $Pw_{i}$ are validated. Otherwise, the session is expired. $SC_{i}$ continues to compute $v_{ij}=s_{ij}⊕H(Id_{i}||RPw_{i})$ and randomly selects a number $a_{i}\in Z_{p}^{*}$ to calculate the following: $X_{i}=a_{i}P,X_{ij}=a_{i}Q_{j},PId_{i}=H\left(X_{ij}\right)⊕Id_{i},h_{i}=h(v_{ij}||Id_{i}||SId_{j}\left|\right|X_{ij}\left|\right|X_{i}).$ Finally, $U_{i}$ transmits the request $\left\{PId_{i},X_{i},h_{ij}\right\}$ to $S_{j}$ via an open channel.
2. After receiving $\left\{PId_{i},X_{i},h_{ij}\right\}$, $S_{j}$ calculates $X_{ij}^{*}=r_{j}X_{i}$, $Id_{i}^{*}=PId_{i}⊕H\left(X_{ij}^{*}\right)$ and $v_{ij}^{*}=H(r_{j}||Id_{i}^{*}\left|\right|c_{j}).$ Afterwards, $S_{j}$ computes $h_{i}^{*}=h(v_{ij}^{*}||Id_{i}^{*}||SId_{j}\left|\right|X_{ij}^{*}\left|\right|X_{i}).$ Then, $S_{j}$ verifies $h_{i}^{*}\overset{?}{=}h_{i}$. In the case of invalidation, $S_{j}$ terminates the session and sets the counter N=1. $S_{j}$ keeps suspending the card until $U_{i}$ registers again if *N* surpasses some threshold mark (e.g., 8). Otherwise, $S_{j}$ randomly selects a number $b_{j}$ to compute $Y_{j}=b_{j}P,z_{ij}=b_{i}X_{i}$, $SK_{ij}=H(Id_{i}||SId_{j}\left|\right|v_{ij}\left|\right|z_{ij}\left|\right|X_{ij}^{*}),$ and $R_{j}=H(PId_{i}\left|\right|v_{ij}||SK_{ij}\left|\right|X_{i}\left|\right|Y_{j}).$ Finally, $S_{j}$ sends the response message $\left\{Y_{j},R_{j}\right\}$ to $U_{i}$ via open channel.
3. Upon receiving the respond message $\left\{Y_{j},R_{j}\right\}$, $U_{i}$ computes $z_{ij}^{*}=a_{i}Y_{i}$, $SK_{ij}^{*}=H(Id_{i}||SId_{j}\left|\right|v_{ij}\left|\right|z_{ij}^{*}\left|\right|X_{ij}),$ and $R_{j}^{*}=H(PId_{i}\left|\right|v_{ij}||SK_{ij}^{*}\left|\right|X_{i}\left|\right|Y_{j}).$ Subsequently, $U_{i}$ checks whether $R_{j}^{*}\overset{?}{=}R_{j}$. The session is aborted if these are not equal, . Otherwise, $S_{j}$ is authenticated by $U_{i}$ and $U_{i}$ accepts $SK_{ij}^{*}$. Afterwards, $U_{i}$ computes $R_{i}=H(v_{ij}\left|\right|X_{i}\left|\right|Y_{j}||SK_{ij}^{*}||Id_{i}).$ Finally, $U_{i}$ transmits the challenge message $R_{i}$ to $S_{j}$ through an open channel.
4. Upon receiving the challenge message from $U_{i}$, $S_{j}$ computes $R_{i}^{*}=H(v_{ij}\left|\right|X_{i}\left|\right|Y_{j}||SK_{ij}^{*}||Id_{i})$ and verifies whether $R_{i}^{*}\overset{?}{=}R_{i}$. If these are equal, then $U_{i}$ is authenticated successfully.

Finally, both $U_{i}$ and $S_{j}$ share the common session key $SK=SK_{ij}^{*}=SK_{ij}$.

### 7.5. Password Updation

$U_{i}$ is able to change their password whenever they want, for which $U_{i}$ and $SC_{i}$ have to undergo the following procedure:

1. $U_{i}$ inserts the $SC_{i}$ into a card reader and inputs $Id_{i}$, current password $Pw_{i}$, and password to be updated $Pw_{i}^{*}$.
2. $SC_{i}$ computes $RPw_{i}=H(Id_{i}||Pw_{i}\left|\right|r_{i}),$ and $A_{i}^{\prime }=H\left(\left(H\left(Id_{i}\right)⊕RPw_{i}\right)\;\mathrm{mod}\;n_{0}\right).$ Afterwards, $SC_{i}$ checks whether $A_{i}^{\prime }\overset{?}{=}A_{i}$. In case of inequality, $SC_{i}$ refuses $U_{i}$ to update the password.
3. Apart from that, $SC_{i}$ randomly selects a number $r_{i}^{*}$ to compute $RPw_{i}^{*}=H(Id_{i}||Pw_{i}^{*}\left|\right|r_{i}^{*}),$$s_{ij}^{*}=s_{ij}⊕H(Id_{i}||RPw_{i}^{*})⊕H(Id_{i}||RPw_{i}^{*}).$ Subsequently, $SC_{i}$ computes $A_{i}^{*}=H(\left(H\left(Id_{i}\right)⊕RPw_{i}^{*}\right)$$\mathrm{mod}\;n_{0}).$ Finally, $SC_{i}$ replaces $r_{i},A_{i},s_{ij}$ with $r_{i}^{*},A_{i}^{*},s_{ij}^{*}$, respectively.

Remark: As Amin et al.’s scheme and Srinivas et al.’s scheme are vulnerable to offline password guessing attack and key-compromise user impersonation attack and cannot provide user un-traceability, and because Amin et al.’s scheme cannot provide perfect forward secrecy, in the proposed scheme: (1) we employ “honey words” + “fuzzy-verifiers” to resist against offline password guessing attack [42]; (2) according to [47], to provide perfect forward secrecy, we use public key cryptosystems (e.g., ECC); (3) we store a secret parameter $c_{j}$ in the server database which cannot be compromised by the adversary in order to resist key-compromise user impersonation attack; and (4) to provide user un-traceability, we deploy a dynamic identity technique via a public key algorithm, that is, $PId_{i}$.

## 8. Security Inspection

This section provides the details of how the presented protocol ensures the security against all known attacks, including key-compromise user impersonation attack and offline password guessing attack. Further, it also offers more comprehensive security features, in particular, user un-traceability and perfect forward secrecy under the capabilities of the adversary that were introduced in Section 2.3.

### 8.1. User Un-Traceability and Anonymity

During the login authentication stage, $Id_{i}$ is not sent through the public channel. Even if A intercepts the login request messages $\left\{PID_{i},X_{i},h_{ij}\right\}$ from the public channel, A still cannot extract $Id_{i}$ from $PId_{i}$, because $PId_{i}$ is protected by $H(X_{ij})$ and is a dynamic identity. Thus, the proposed scheme provides the user un-traceability and anonymity.

### 8.2. Stolen Smart-Card Attack

In the proposed scheme, even if A steals $SC_{i}$ of $U_{i}$, then A can extract the parameters $\left\{A_{i},r_{i},s_{ij}\left(1\leq j\leq k\right),n_{0},Q_{j},P,H\left(\cdot \right)\right\}$ stored in $SC_{i}$ utilizing power analysis technology, and captures the transmitted message over a public channel. However, as per the following details, A cannot execute any attack. Thus, the presented protocol is secured against stolen smart-card attack.

### 8.3. Offline Password Guessing Attack

Assuming that A steals $SC_{i}$ and extracts $\left\{A_{i},r_{i},s_{ij}\left(1\leq j\leq k\right),n_{0},Q_{j},P,H\left(\cdot \right)\right\}$ stored in it. A intercepts all messages $\left\{PId_{i},X_{i},h_{ij}\right\},\left\{Y_{j},R_{j}\right\},\left\{R_{i}\right\}$ over a public channel. If A guesses an ID $Id_{i}^{\prime }$ and a password $Pw_{i}^{\prime }$, A can calculate $RPw_{i}^{\prime }=H(Id_{i}^{\prime }||Pw_{i}^{\prime }\left|\right|r_{i})$, and then figures out $A_{i}^{\prime }=H\left(\left(H\left(Id_{i}\right)⊕RPw_{i}^{\prime }\right)\;\mathrm{mod}\;n_{0}\right)$. Afterwards, A examines whether $A_{i}^{\prime }\overset{?}{=}A_{i}$. According to [42], A can obtain the reduced password guessing space of size $\frac{\left|D\right|}{n_{0}}$, where D is the space of passwords. Further, A can guess the correct password only by online password guessing. However, $S_{j}$ prevents this guessing by using a login request threshold value (e.g., 8). Once the number of online guesses exceeds the threshold value, $S_{j}$ will terminate communication and suspend $SC_{i}$ until $U_{i}$ registers again. Therefore, the presented scheme offers resistance against offline password guessing attack.

### 8.4. Privileged Insider Attack

If an internal attacker eavesdrops the registration information $\left\{Id_{i},RPw_{i}\right\}$ during user registration, A is unable to get $Pw_{i}$, because it is secured by one-way hash function H(·) as well as with random number $r_{i}$. Thus, the presented scheme is immune to the privileged insider attack.

### 8.5. Key-Compromise User Impersonation Attack

If the adversary steals the long-term private key of the server, it is still unable to impersonate the user to the server. This kind of attack is referred to as a key-compromise user impersonation attack. In the presented protocol, even if $r_{j}$ of $S_{j}$ is revealed to A, still A cannot determine $v_{ij}=H(r_{j}||Id_{i}\left|\right|c_{j})$, because A is unable to obtain the random number $c_{j}$. Therefore, A cannot forge the login request message $\left\{h_{ij}\right\}$, and therefore cannot be authenticated by $S_{j}$. That is, A cannot impersonate $U_{i}$. Thus, the presented protocol is insusceptible to key-compromise user impersonation attack. Further, it implies that the presented scheme ensures resistance against user impersonation attack.

### 8.6. Server Impersonation Attack

A intercepts the response message $\left\{Y_{j},R_{j}\right\}$ if A tries to make a server impersonation attack. A randomly generates a number $b_{j}^{\prime }$ to compute $Y_{j}^{\prime }=b_{j}^{\prime }P,z_{ij}^{\prime }=b_{j}^{\prime }X_{i}$. Afterwards, A tries to compute $SK_{ij}^{\prime }$ and $R_{j}^{\prime }$. Since A does not know $v_{ij}$ and $X_{ij}^{*}$ computed by the secret key $\left\{r_{j},c_{j}\right\}$ of $S_{j}$, A is unable to calculate $SK_{ij}^{\prime }$ and cannot forge $R_{j}$. Thus, A cannot carry out the server impersonation attack.

### 8.7. Replay Attack

If A intercepts the login message $\left\{PId_{i},X_{i},h_{ij}\right\}$ from $U_{i}$, and wants to replay this message to $S_{j}$. This replay attack is easily captured by inspecting the freshness of $X_{i}$ in the presented scheme, where $X_{i}=a_{i}P$, and $a_{i}$ is a random number. Similarly, replaying the challenging message and response message is detected by either $U_{i}$ or $S_{j}$. Thereupon, it is inferred that the presented protocol is immune to replay attack.

### 8.8. Known Key Security

Suppose that A compromises the previous session key $SK_{ij}=H(Id_{i}||SId_{j}\left|\right|v_{ij}\left|\right|z_{ij}\left|\right|X_{ij})$ between $U_{i}$ and $S_{j}$. However, the next session key $SK_{ij}^{\prime }$ will be computed by new random numbers $a_{i}^{\prime }$ and $b_{j}^{\prime }$. That is, $SK_{ij}^{\prime }=H(Id_{i}||SId_{j}\left|\right|v_{ij}\left|\right|z_{ij}^{\prime }\left|\right|X_{ij}^{\prime })$. To calculate the new session key, A has to compute $a_{i}^{\prime },b_{j}^{\prime },a_{i}^{\prime }b_{j}^{\prime }P,a_{i}^{\prime }Y_{j},b_{j}^{\prime }X_{i}$ from $X_{i}^{\prime },Y_{j}^{\prime }$. However, this is computationally infeasible for A because of ECDLP and ECCDHP. Therefore, the presented scheme offers known key security.

### 8.9. Mutual Authentication

In the proposed scheme, only the legitimate $h_{ij}$ and $R_{i}$ can be verified by $S_{j}$, and only the legitimate $R_{j}$ can be verified as the user $U_{i}$. That is, the proposed scheme allows $S_{j}$ and $U_{j}$ to authenticate each other. Thus, the presented protocol ensures mutual authentication between a legitimate $U_{i}$ and $S_{j}$.

### 8.10. Man-in-the-Middle Attack

It is impossible for A in the proposed scheme to compute the correct login request and challenge message. Therefore, A cannot be authenticated by the server. Moreover, A is unable to calculate the correct response message, and thus A cannot pass the user verification. It is therefore inferred that the proposed scheme is immune to man-in-the-middle attack.

### 8.11. Denial-of-Service Attack

If $U_{i}$ wants the login authentication in the proposed scheme, it must input the correct $Id_{i}$ and $Pw_{i}$ to pass the verification of SC. If A inputs wrong $Id_{i}$ and $Pw_{i}$ into SC, A is unable to compute the correct login request message. Moreover, if $U_{i}$ wants to update the password, it has to pass the verification of SC. An incorrect or previous password cannot pass the verification. Therefore, the proposed scheme ensures resistance against denial-of-service attack.

### 8.12. Perfect Forward Secrecy

Suppose that $r_{j}$ of $S_{j}$ is compromised and A acquires $r_{i}$, $Id_{i}$, and $Pw_{i}$. To calculate the correct $SK_{ij}=H(Id_{i}||SId_{j}\left|\right|v_{ij}\left|\right|z_{ij}\left|\right|X_{ij})$, A is required to calculate $z_{ij},X_{ij}$. However, it is impossible for A to compute $z_{ij},X_{ij}$ because of ECDLP and ECCDHP. Thus, A is not capable of figuring out $SK_{ij}$. Therefore, the presented protocol ensures perfect forward secrecy.

## 9. BAN-Logic Proof

BAN is a logic of belief. The intended use of BAN is to analyze authentication protocols by deriving the beliefs that honest principals correctly executing a protocol can come to as a result of the protocol execution. For example, a user might come to believe that a session key they have negotiated with a server is a good key for a future session [51]. This section incorporates the BAN-Logic [52] to prove the session key agreement between user $U_{i}$ and server $S_{j}$ after the execution of the improved scheme. BAN-Logic notations and Basic BAN-Logic postulates are described in Table 2 and Table 3.

### 9.1. Idealized Scheme

The ideal form of the presented protocol is derived as follows:

**Message** **1.** $U_{i}\to S_{j}$: $X_{i}$, $<Id_{i}{>}_{U_{i}\overset{H(X_{ij})}{⟷}S_{j}},{\left(Id_{i},SId_{j},X_{ij},X_{i}\right)}_{U_{i}\overset{v_{ij}}{⟷}S_{j}},{\left(X_{i},Y_{j},U_{i}\overset{SK}{⟷}S_{j},Id_{i}\right)}_{U_{i}\overset{v_{ij}}{⟷}S_{j}}.$
**Message** **2.** $S_{j}\to U_{i}$: $Y_{j}$, ${\left(PId_{i},U_{i}\overset{SK}{⟷}S_{j},X_{i},Y_{j}\right)}_{U_{i}\overset{v_{ij}}{⟷}S_{j}}$.

### 9.2. Security Objectives

We prove that the improved scheme can satisfy the following objective:

**Objective** **1.** $U_{i}\left|\equiv S_{j}\right|\equiv \left(U_{i}\overset{SK}{⟷}S_{j}\right).$
**Objective** **2.** $U_{i}|\equiv \left(U_{i}\overset{SK}{⟷}S_{j}\right).$
**Objective** **3.** $S_{j}\left|\equiv U_{i}\right|\equiv \left(U\overset{SK}{⟷}S_{j}\right).$
**Objective** **4.** $S_{j}|\equiv \left(U_{i}\overset{SK}{⟷}S_{j}\right).$

### 9.3. Initiative Premises

For the initial status of the proposed scheme, the following assumptions are made.

**IP** **1.** $U_{i}|\equiv ♯\left(a_{i}\right)$.
**IP** **2.** $S_{j}|\equiv ♯\left(b_{j}\right)$.
**IP** **3.** $U_{i}|\equiv \left(U_{i}\overset{X_{ij}}{⟷}S_{j}\right)$.
**IP** **4.** $S_{j}|\equiv \left(U_{i}\overset{X_{ij}}{⟷}S_{j}\right)$.
**IP** **5.** $U_{i}|\equiv \left(U_{i}\overset{v_{ij}}{⟷}S_{j}\right)$.
**IP** **6.** $S_{j}|\equiv \left(U_{i}\overset{v_{ij}}{⟷}S_{j}\right)$.
**IP** **7.** $U_{i}|\equiv S_{j}\Rightarrow \left(U_{i}\overset{SK}{⟷}S_{j}\right)$.
**IP** **8.** $S_{j}|\equiv U_{i}\Rightarrow \left(U_{i}\overset{SK}{⟷}S_{j}\right)$.

### 9.4. Proof Procedure

The main proof steps of the proposed scheme are presented below.

**Step** **1.** From Message 2, it shows the following:
  $$U_{i}⊲{\left(PId_{i},U_{i}\overset{SK}{⟷}S_{j},X_{i},Y_{j}\right)}_{U_{i}\overset{v_{ij}}{⟷}S_{j}}.$$
**Step** **2.** From Step 1, IP 5, and the message-meaning rule, it illustrates the following:
  $$U_{i}\left|\equiv S_{j}\right|∼\left(PId_{i},U_{i}\overset{SK}{⟷}S_{j},X_{i},Y_{j}\right).$$
**Step** **3.** From IP 1 and the freshness conjuncatenation rule, the following can be inferred:
  $$U_{i}|\equiv ♯\left(PId_{i},U_{i}\overset{SK}{⟷}S_{j},X_{i},Y_{j}\right).$$
**Step** **4.** From Steps 2 and 3, the freshness rule, and the nonce verification rule, we obtain the following:
  $$U_{i}\left|\equiv S_{j}\right|\equiv \left(PId_{i},U_{i}\overset{SK}{⟷}S_{j},X_{i},Y_{j}\right).$$
**Step** **5.** From Step 4 and the believe rule, we deduce the first objective as follows:
  $$U_{i}\left|\equiv S_{j}\right|\equiv \left(U_{i}\overset{SK}{⟷}S_{j}\right)\;\;\;\;\left(\mathrm{Objective}\;1\right).$$
**Step** **6.** From Objective 1, IP 7, and the jurisdiction rule, we accomplish the second objective as follows:
  $$U_{i}|\equiv \left(U_{i}\overset{SK}{⟷}S_{j}\right)\;\;\;\;\left(\mathrm{Objective}\;2\right).$$
**Step** **7.** From Message 1, it indicates the following:
  $$S_{j}⊲{\left(X_{i},Y_{j},U_{i}\overset{SK}{⟷}S_{j},Id_{i}\right)}_{U_{i}\overset{v_{ij}}{⟷}S_{j}}.$$
**Step** **8.** From Step 7, IP 6, and the message meaning rule, the following can be inferred:
  $$S_{j}\left|\equiv U_{i}\right|∼\left(X_{i},Y_{j},U_{i}\overset{SK}{⟷}S_{j},Id_{i}\right).$$
**Step** **9.** From IP 2 and the freshness conjuncatenation rule, the following can be obtained:
  $$S_{j}|\equiv ♯\left(X_{i},Y_{j},U_{i}\overset{SK}{⟷}S_{j},Id_{i}\right).$$
**Step** **10.** From Steps 8 and 9, the freshness rule, and the nonce-verification rule, we determine the following:
  $$S_{j}\left|\equiv U_{i}\right|\equiv \left(X_{i},Y_{j},U_{i}\overset{SK}{⟷}S_{j},Id_{i}\right).$$
**Step** **11.** From Step 10 and the believe rule, the third objective can be achieved as follows:
  $$S_{j}\left|\equiv U_{i}\right|\equiv U_{i}\overset{SK}{⟷}S_{j}\;\;\;\;\left(\mathrm{Objective}\;3\right).$$
**Step** **12.** From Objective 3, IP 8, and the jurisdiction rule, the fourth objective is accomplished as follows:
  $$S_{j}|\equiv \left(U_{i}\overset{SK}{⟷}S_{j}\right)\;\;\;\;\left(\mathrm{Objective}\;4\right).$$

By accomplishing Objectives 1–4, both $U_{i}$ and $S_{j}$ believe that the SK is settled between them. Therefore, the proposed scheme ensures mutual authentication along with key agreement.

## 10. Performance Comparison

This section analyzes the computational and security performance of the presented scheme while comparing it with multiple schemes, including those of Awasthi et al. [23], Huang et al. [25], Amin et al. [26], Pippal et al. [27], Li et al. [28], and Srinivas et al. [29]. The exclusive-OR operation and string concatenation are usually neglected when comparing the computational cost. However, the following operations are considered: $T_{me}$, the execution time of point multiplication operation; $T_{e}$, the time for execution of modular exponentiation operation; $T_{h}$, the running time of a hash operation; and $T_{mm}$, the running time for modular multiplication operation. More precisely, we compare the experimental results of the aforementioned operations as performed by [53,54], where $T_{e}$, $T_{me}$, $T_{h}$, and $T_{mm}$ take 3.85 ms, 2.226 ms, 0.0023 ms, and 0.001855 ms, respectively (Table 4). Following [53,54], the aforementioned operations were executed on a computing platform having Intel Pentium Dual Core E2200 2.20 GHz processor, the Ubuntu 12.04.1 LTS 32-bits operating system, and 2048 MB of RAM.

In Table 5, we compare the schemes of [23,25,26,27,28,29] with the presented protocol in terms of security. In Table 5, we observe that [23,25,26,27,28,29] cannot provide $\left[C_{1}-C_{3},C_{5}\right]$ features. The scheme in [26] is still unable to provide perfect forward secrecy $\left[C_{12}\right]$, although the authors used RSA-based public cryptography. The proposed scheme fulfills all known security features $\left[C_{1}-C_{12}\right]$. Thus, the presented scheme surpasses [23,25,26,27,28,29] in terms of security.

Table 6 presents the computational cost of the schemes [23,25,26,27,28,29] and the proposed scheme for login and authentication. The computational cost of the proposed protocol is comparatively lower than the schemes in [23,25,27,28,29], but slightly higher than the scheme in [26]. However, according to Table 5, the scheme in [26] cannot address $\left[C_{1}-C_{3},C_{5},C_{12}\right]$ security features. Thus, combining Table 5 and Table 6, we remark that the presented solution is more feasible for practical multi-server environments in terms of the trade-off between usability and security.

## 11. Conclusions

This paper first analyzes Amin et al.’s [26] scheme and proves that the considered scheme cannot provide perfect forward secrecy and user un-traceability, and is susceptible to offline password guessing attack and key-compromise user impersonation attack. Second, we review Srinivas et al.’s [29] multi-server authentication scheme while proving that it cannot resist offline password guessing attack and key-compromise user impersonation attack, and is unable to ensure user un-traceability. Afterwards, to address the limitations of prevalent works, we put forward an enhanced multi-server two-factor authentication scheme. Heuristic analysis and BAN-Logic proof ensure that the presented scheme includes various known security features. The security and efficiency analyses display the robustness and efficiency of the presented scheme. Overall, the presented scheme is proven to be more feasible for multi-server authentication-and-key-agreement scenarios in various low-power networks. Moreover, the design and analysis methods in this paper can also be used for authentication protocols in IoT, WSNs, etc.

## Figures and Tables

**Table 1:** Notations and their descriptions.

Symbol	Description	Symbol	Description
RC	Registration center	$S_{j}$	Server
$U_{i}$	User	$SC_{i}$	Smart card of $U_{i}$
$Id_{i}$	Identification of user $U_{i}$	$Pw_{i}$	Password belonging to user $U_{i}$
$r_{i},a_{i}$	Random numbers of $U_{i}$	*p*	Large prime
$Q_{j}=r_{j}P$	Public key of $S_{j}$	$r_{j}$	Private key of $S_{j}$
$c_{j},b_{j}$	Random number of $S_{j}$	⊕	The bitwise XOR operation
||	The string concatenation operation	H(·)	One-way hash function
A	The malicious adversary	$SK_{ij}$	Session key belonging to $U_{i}$ and $S_{j}$

**Table 2:** Burrows–Abadi–Needham logic (BAN-Logic) notations.

Symbol	Description
A|≡X	*A* has trust on *X*
A⊲X	*A* acquires/observes *X*
A|∼X	*A* sends *X* *X* (or *A* once called)
A|⇒X	*A* regulates *X*
♯(X)	*X* is fresh
$A\overset{K}{⟷}B$	*A* and *B* utilize shared key *K* for communication
${\left(X,Y\right)}_{K}$	use *K* as key to compute hash values of *X* and *Y*
$<X{>}_{K}$	*X* is exclusive or-ed with *K*

**Table 3:** Basic BAN-Logic postulates

Rule	Description
Message−meaningrule	$\frac{A|\equiv A\overset{K}{⟷}B,A⊲{\left(X\right)}_{K}}{A|\equiv B|∼X}$
Nonceverificationrule	$\frac{A|\equiv ♯(X),A|\equiv B|∼X}{A|\equiv B|\equiv X}$
Jurisdictionrule	$\frac{A|\equiv B|\Rightarrow X,A|\equiv B|\equiv X}{A|\equiv X}$
Freshnessconjuncatenationrule	$\frac{A|\equiv ♯(X)}{A|\equiv ♯(X,Y)}$
Believerule	$\frac{A|\equiv B|\equiv (X,Y)}{A|\equiv B|\equiv X}$, $\frac{A|\equiv X,A|\equiv Y}{A|\equiv (X,Y)}$

**Table 4:** The performing time of cryptographic operations (adapted from [53,54]).

Symbol	$T_{e}$	$T_{\mathrm{me}}$	$T_{h}$	$T_{\mathrm{mm}}$
Time	3.85 ms	2.226 ms	0.0023 ms	0.001855 ms

**Table 5:** Comparison of security features.

	Schemes	Awasthi et al. [23]	Huang et al. [25]	Amin et al. [26]	Pippal et al. [27]	Li et al. [28]	Srinivas et al. [29]	Proposed Scheme
Features								
$C_{1}$		No	No	No	No	No	No	Yes
$C_{2}$		No	No	No	No	No	No	Yes
$C_{3}$		No	No	No	No	No	No	Yes
$C_{4}$		No	No	Yes	No	No	Yes	Yes
$C_{5}$		No	No	No	No	No	No	Yes
$C_{6}$		No	Yes	Yes	No	No	Yes	Yes
$C_{7}$		Yes	Yes	Yes	Yes	Yes	Yes	Yes
$C_{8}$		N/A	N/A	Yes	Yes	Yes	Yes	Yes
$C_{9}$		Yes	Yes	Yes	Yes	Yes	Yes	Yes
$C_{10}$		No	Yes	Yes	No	No	Yes	Yes
$C_{11}$		No	No	Yes	No	No	Yes	Yes
$C_{12}$		N/A	N/A	No	Yes	Yes	Yes	Yes

$C_{1}$ provides user anonymity and un-traceability. $C_{2}$ resists stolen smart-card attack. $C_{3}$ resists offline password guessing attack. $C_{4}$ resists privileged insider attack. $C_{5}$ resists (key-compromised) user impersonation attack. $C_{6}$ resists server-impersonation attack. $C_{7}$ resists replay attack. $C_{8}$ provides known key security. $C_{9}$ provides mutual authentication. $C_{10}$ resists man-in-the-middle attack. $C_{11}$ resists denial-of-service attack. $C_{12}$ provides perfect forward secrecy.

**Table 6:** Comparison of computational complexity.

	Cost	User Computation	Server Computation	Total
Schemes				
Awasthi et al. [23]		$3T_{e}+3T_{mm}+2T_{h}$	$3T_{e}+T_{mm}+3T_{h}$	$6T_{e}+4T_{mm}+5T_{h}\approx 23.1189$ ms
Huang et al. [25]		$2T_{e}+2T_{h}$	$3T_{e}+3T_{h}$	$5T_{e}+5T_{h}\approx 19.2615$ ms
Amin et al. [26]		$T_{e}+6T_{h}$	$T_{e}+4T_{h}$	$2T_{e}+10T_{h}\approx 7.723$ ms
Pippal et al. [27]		$3T_{e}+T_{mm}+4T_{h}$	$4T_{e}+T_{mm}+3T_{h}$	$7T_{e}+2T_{mm}+7T_{h}\approx 26.9698$ ms
Li et al. [28]		$T_{e}+5T_{h}$	$3T_{e}+8T_{h}$	$4T_{e}+13T_{h}\approx 15.4299$ ms
Srinivas et al. [29]		$2T_{e}+8T_{h}$	$2T_{e}+4T_{h}$	$4T_{e}+12T_{h}\approx 15.676$ ms
Proposed scheme		$3T_{me}+9T_{h}$	$3T_{me}+6T_{h}$	$6T_{me}+15T_{h}\approx 13.3905$ ms
